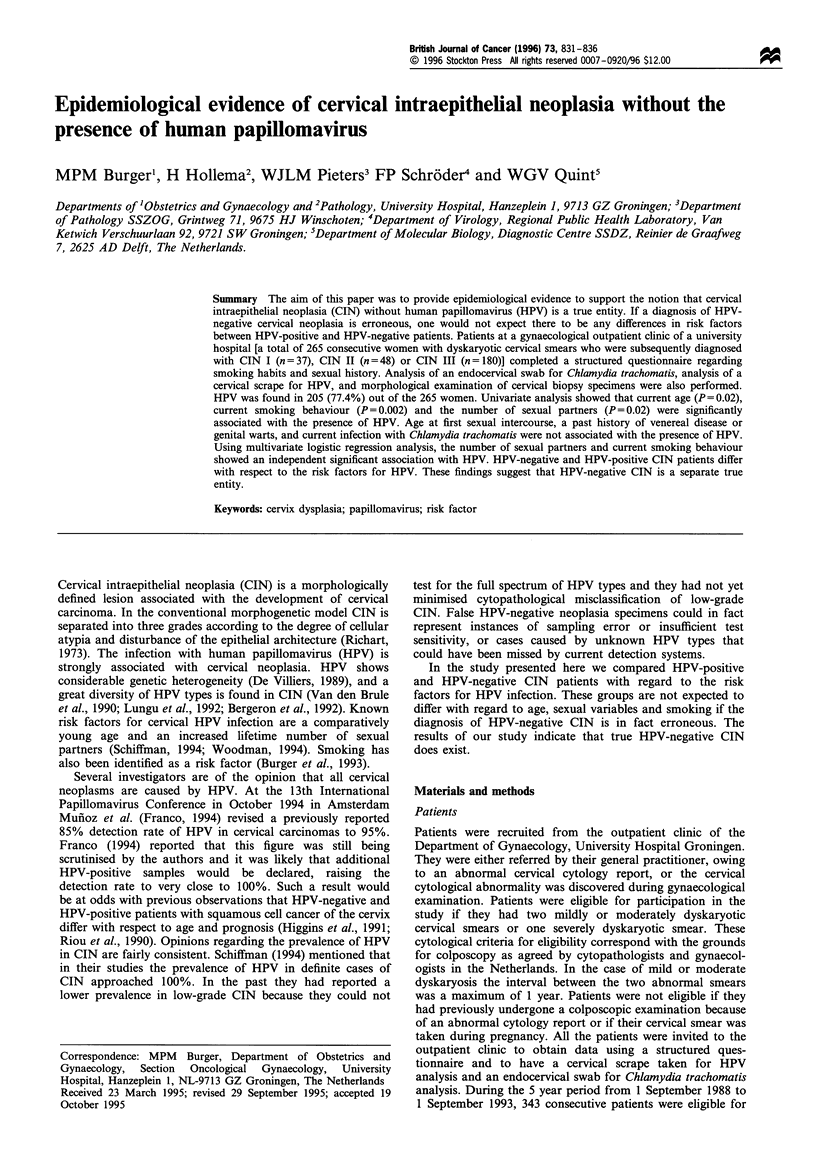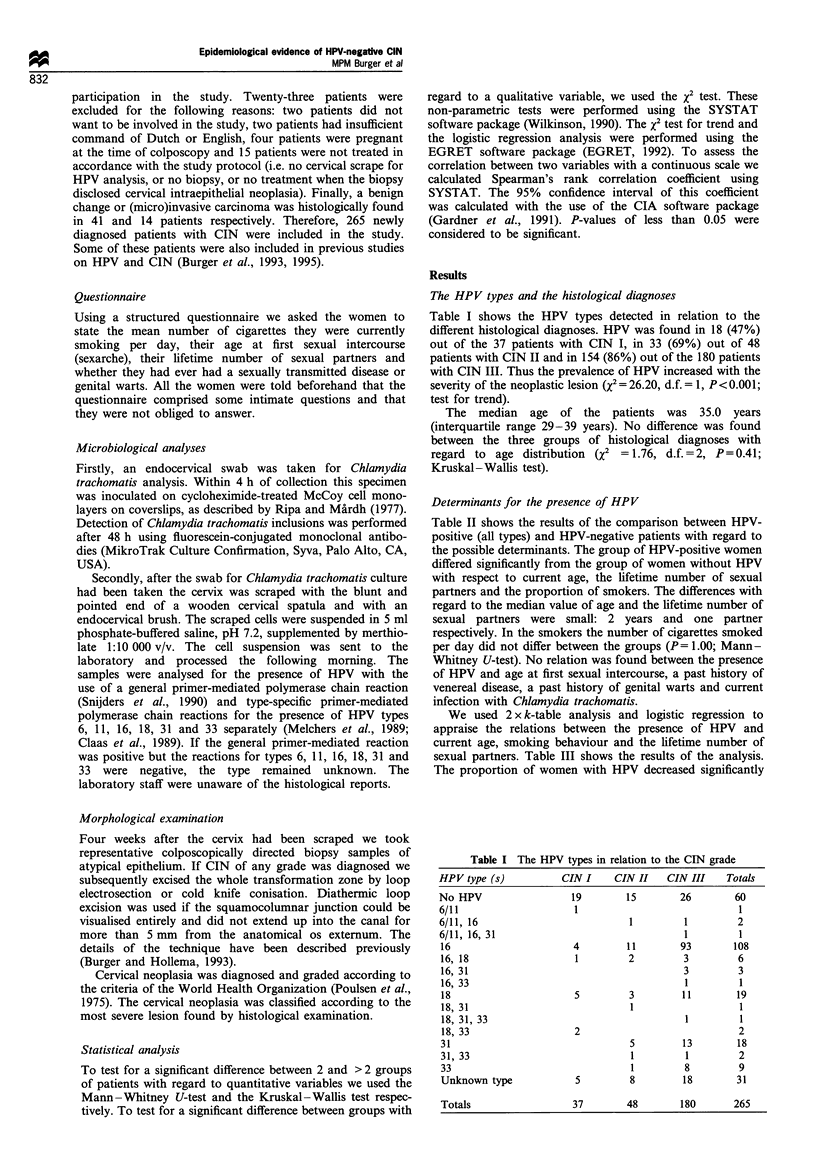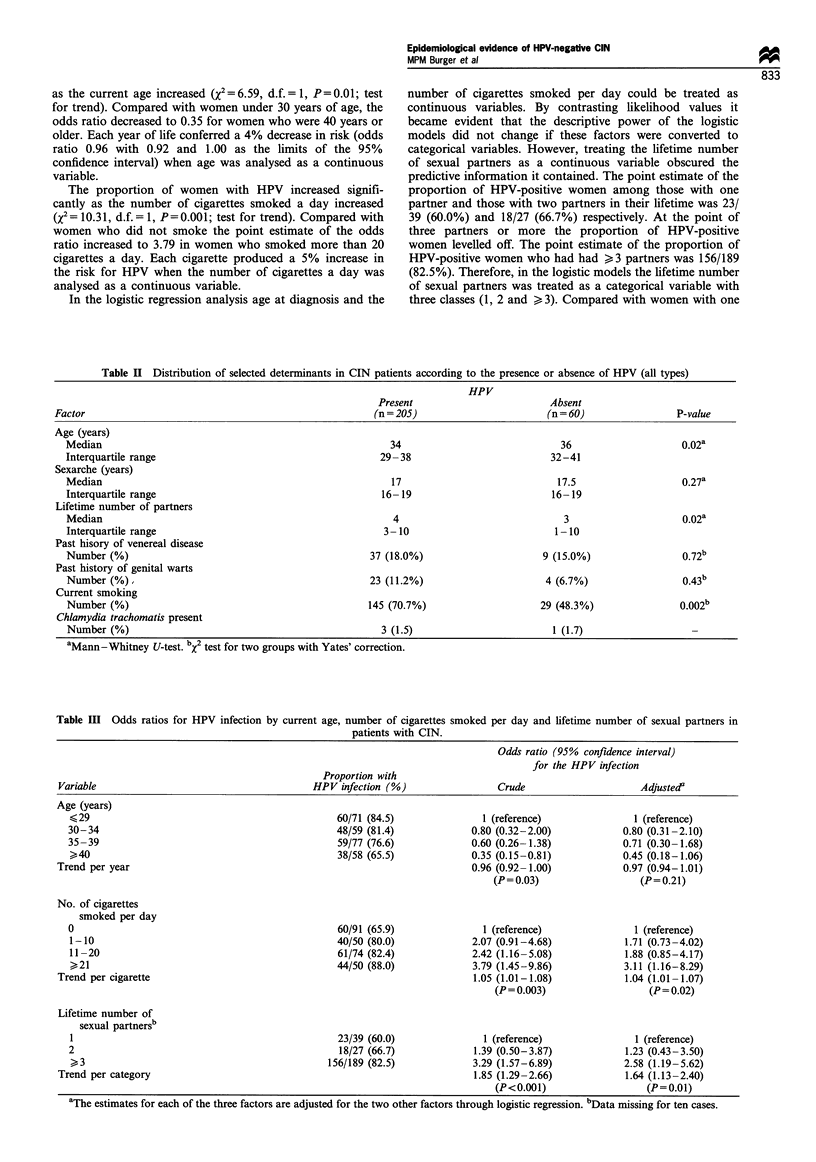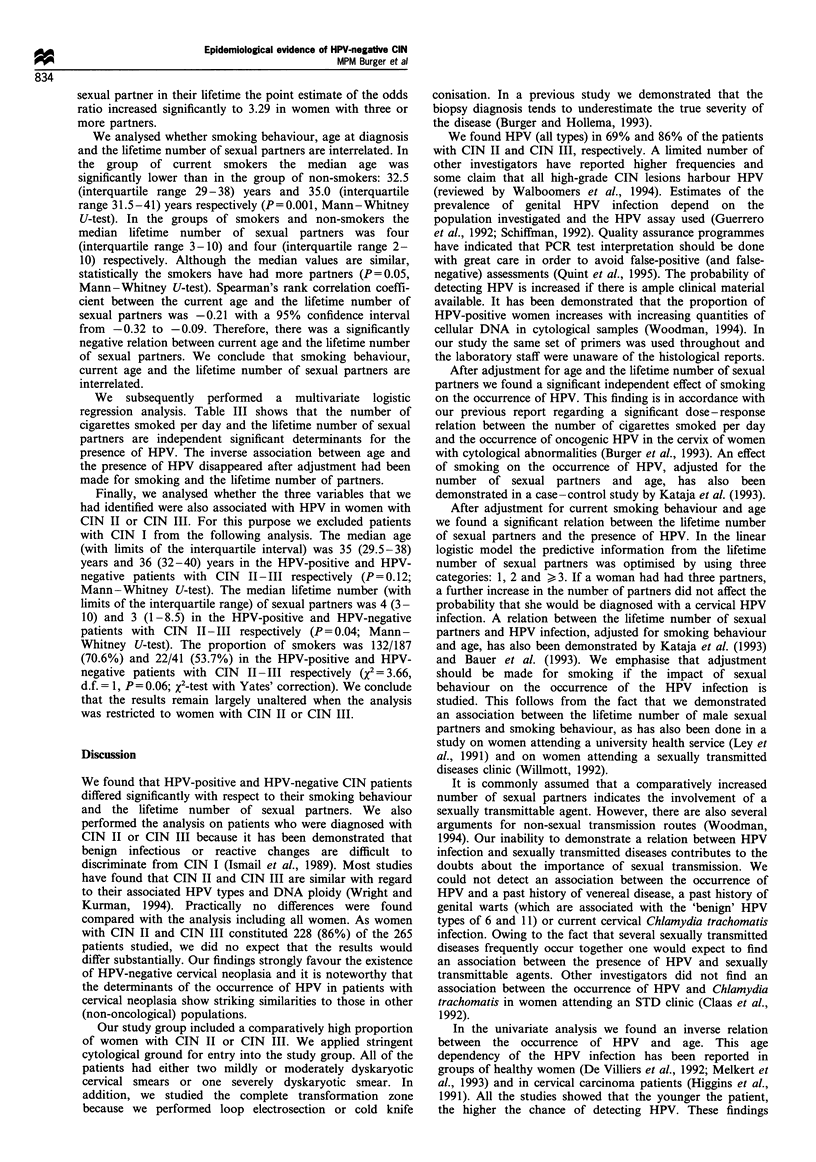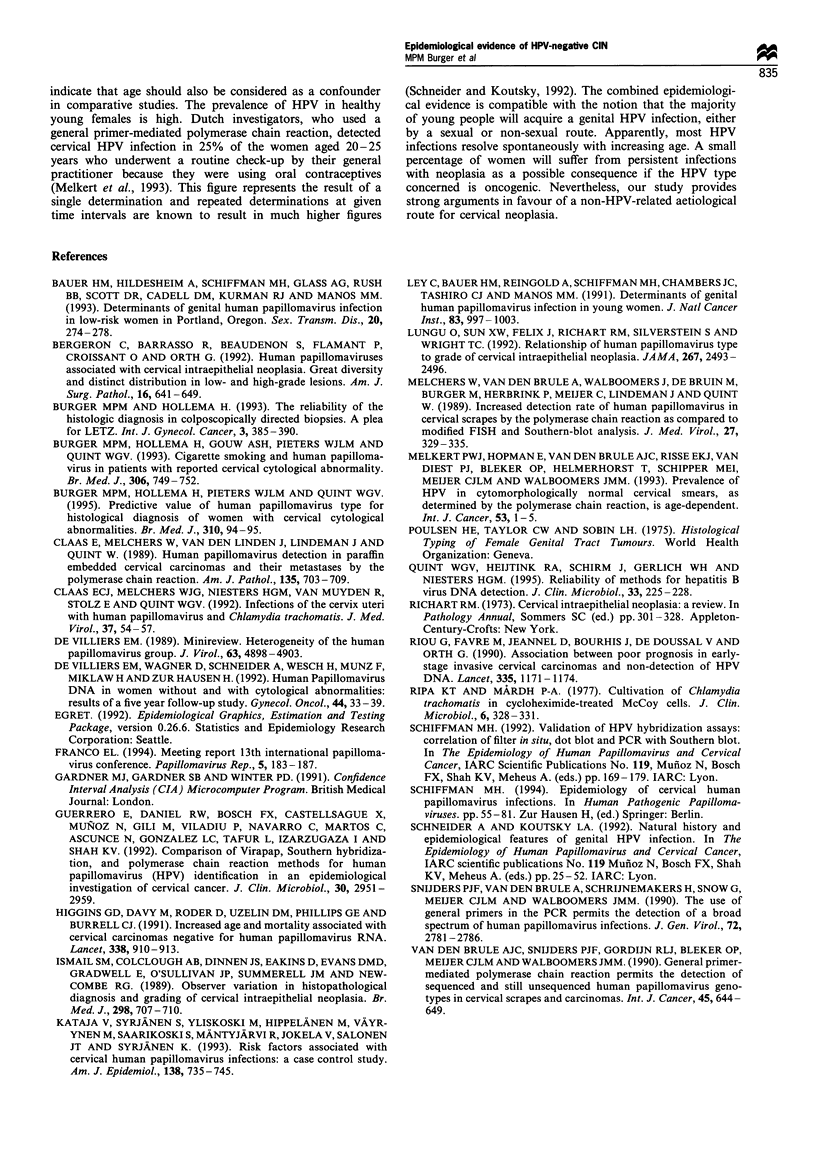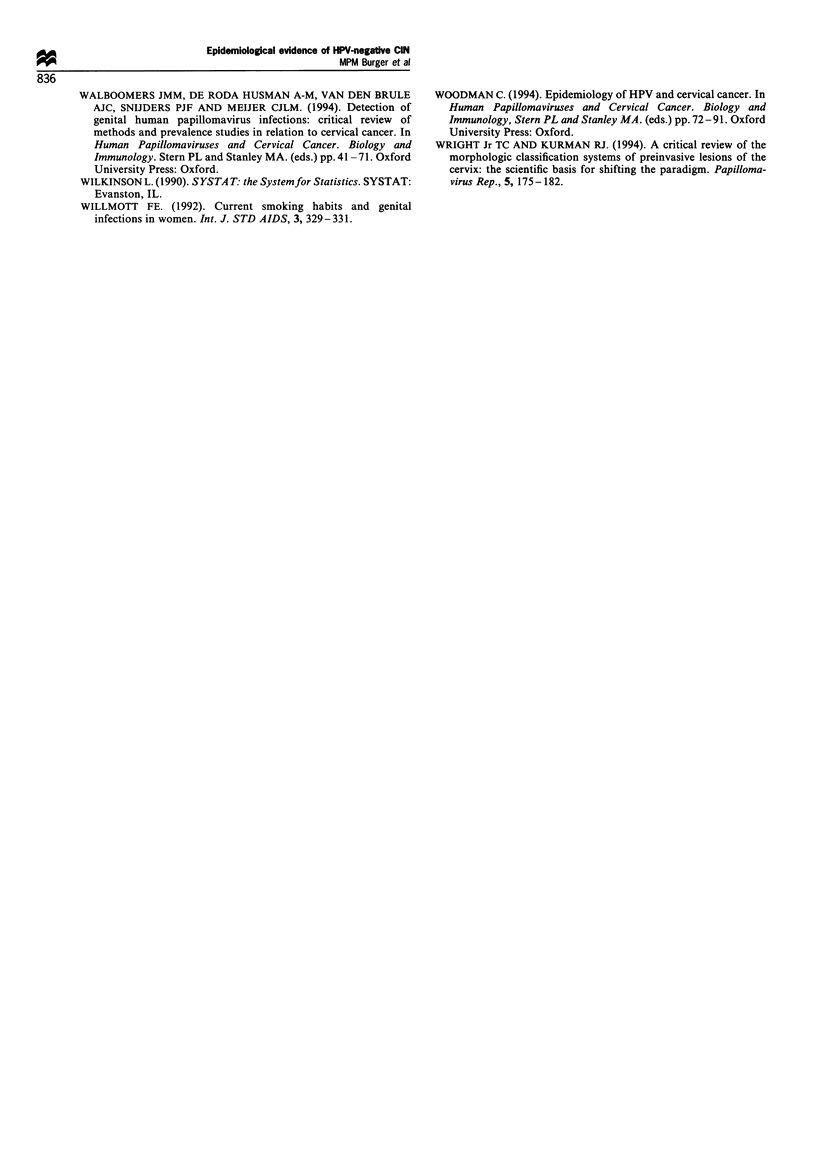# Epidemiological evidence of cervical intraepithelial neoplasia without the presence of human papillomavirus.

**DOI:** 10.1038/bjc.1996.146

**Published:** 1996-03

**Authors:** M. P. Burger, H. Hollema, W. J. Pieters, F. P. Schröder, W. G. Quint

**Affiliations:** Department of Obstetrics and Gynaecology, University Hospital, Groningen, The Netherlands.

## Abstract

The aim of this paper was to provide epidemiological evidence to support the notion that cervical intraepithelial neoplasia (CIN) without human papillomavirus (HPV) is a true entity. If a diagnosis of HPV-negative cervical neoplasia is erroneous, one would not expect there to be any differences in risk factors between HPV-positive and HPV-negative patients. Patients at a gynaecological outpatient clinic of a university hospital [a total of 265 consecutive women with dyskaryotic cervical smears who were subsequently diagnosed with CIN I (n=37), CIN II (n=48) or CIN III (n=180)] completed a structured questionnaire regarding smoking habits and sexual history. Analysis of an endocervical swab for Chlamydia trachomatis, analysis of a cervical scrape for HPV, and morphological examination of cervical biopsy specimens were also performed. HPV was found in 205 (77.4%) out of the 265 women. Univariate analysis showed that current age (P=0.02), current smoking behaviour (P=0.002) and the number of sexual partners (P=0.02) were significantly associated with the presence of HPV. Age at first sexual intercourse, a past history of venereal disease or genital warts, and current infection with Chlamydia trachomatis were not associated with the presence of HPV. Using multivariate logistic regression analysis, the number of sexual partners and current smoking behaviour showed an independent significant association with HPV. HPV-negative and HPV-positive CIN patients differ with respect to the risk factors for HPV. These findings suggest that HPV-negative CIN is a separate true entity.


British Journal of Cancer (1996) 73, 831-836
© 1996 Stockton Press All rights reserved 0007-0920/96 $12.00

### Epidemiological evidence of cervical intraepithelial neoplasia without the presence of human papillomavirus

MPM Burger[1], H Hollema[2], WJLM Pieters[3] FP Schröder[4] and WGV Quint[5]

*Departments of [1]Obstetrics and Gynaecology and [2]Pathology, University Hospital, Hanzeplein 1, 9713 GZ Groningen; [3]Department of Pathology SSZOG, Grintweg 71, 9675 HJ Winschoten; [4]Department of Virology, Regional Public Health Laboratory, Van Ketwich Verschuurlaan 92, 9721 SW Groningen; [5]Department of Molecular Biology, Diagnostic Centre SSDZ, Reinier de Graafweg 7, 2625 AD Delft, The Netherlands.*

**Summary** The aim of this paper was to provide epidemiological evidence to support the notion that cervical intraepithelial neoplasia (CIN) without human papillomavirus (HPV) is a true entity. If a diagnosis of HPV-negative cervical neoplasia is erroneous, one would not expect there to be any differences in risk factors between HPV-positive and HPV-negative patients. Patients at a gynaecological outpatient clinic of a university hospital [a total of 265 consecutive women with dyskaryotic cervical smears who were subsequently diagnosed with CIN I ($n$=37), CIN II ($n$=48) or CIN III ($n$=180)] completed a structured questionnaire regarding smoking habits and sexual history. Analysis of an endocervical swab for *Chlamydia trachomatis*, analysis of a cervical scrape for HPV, and morphological examination of cervical biopsy specimens were also performed. HPV was found in 205 (77.4%) out of the 265 women. Univariate analysis showed that current age ($P$=0.02), current smoking behaviour ($P$=0.002) and the number of sexual partners ($P$=0.02) were significantly associated with the presence of HPV. Age at first sexual intercourse, a past history of venereal disease or genital warts, and current infection with *Chlamydia trachomatis* were not associated with the presence of HPV. Using multivariate logistic regression analysis, the number of sexual partners and current smoking behaviour showed an independent significant association with HPV. HPV-negative and HPV-positive CIN patients differ with respect to the risk factors for HPV. These findings suggest that HPV-negative CIN is a separate true entity.

**Keywords:** cervix dysplasia; papillomavirus; risk factor

Cervical intraepithelial neoplasia (CIN) is a morphologically defined lesion associated with the development of cervical carcinoma. In the conventional morphogenetic model CIN is separated into three grades according to the degree of cellular atypia and disturbance of the epithelial architecture (Richart, 1973). The infection with human papillomavirus (HPV) is strongly associated with cervical neoplasia. HPV shows considerable genetic heterogeneity (De Villiers, 1989), and a great diversity of HPV types is found in CIN (Van den Brule *et al.*, 1990; Lungu *et al.*, 1992; Bergeron *et al.*, 1992). Known risk factors for cervical HPV infection are a comparatively young age and an increased lifetime number of sexual partners (Schiffman, 1994; Woodman, 1994). Smoking has also been identified as a risk factor (Burger *et al.*, 1993).

Several investigators are of the opinion that all cervical neoplasms are caused by HPV. At the 13th International Papillomavirus Conference in October 1994 in Amsterdam Muñoz *et al.* (Franco, 1994) revised a previously reported 85% detection rate of HPV in cervical carcinomas to 95%. Franco (1994) reported that this figure was still being scrutinised by the authors and it was likely that additional HPV-positive samples would be declared, raising the detection rate to very close to 100%. Such a result would be at odds with previous observations that HPV-negative and HPV-positive patients with squamous cell cancer of the cervix differ with respect to age and prognosis (Higgins *et al.*, 1991; Riou *et al.*, 1990). Opinions regarding the prevalence of HPV in CIN are fairly consistent. Schiffman (1994) mentioned that in their studies the prevalence of HPV in definite cases of CIN approached 100%. In the past they had reported a lower prevalence in low-grade CIN because they could not test for the full spectrum of HPV types and they had not yet minimised cytopathological misclassification of low-grade CIN. False HPV-negative neoplasia specimens could in fact represent instances of sampling error or insufficient test sensitivity, or cases caused by unknown HPV types that could have been missed by current detection systems.

In the study presented here we compared HPV-positive and HPV-negative CIN patients with regard to the risk factors for HPV infection. These groups are not expected to differ with regard to age, sexual variables and smoking if the diagnosis of HPV-negative CIN is in fact erroneous. The results of our study indicate that true HPV-negative CIN does exist.

### Materials and methods

### *Patients*

Patients were recruited from the outpatient clinic of the Department of Gynaecology, University Hospital Groningen. They were either referred by their general practitioner, owing to an abnormal cervical cytology report, or the cervical cytological abnormality was discovered during gynaecological examination. Patients were eligible for participation in the study if they had two mildly or moderately dyskaryotic cervical smears or one severely dyskaryotic smear. These cytological criteria for eligibility correspond with the grounds for colposcopy as agreed by cytopathologists and gynaecologists in the Netherlands. In the case of mild or moderate dyskaryosis the interval between the two abnormal smears was a maximum of 1 year. Patients were not eligible if they had previously undergone a colposcopic examination because of an abnormal cytology report or if their cervical smear was taken during pregnancy. All the patients were invited to the outpatient clinic to obtain data using a structured questionnaire and to have a cervical scrape taken for HPV analysis and an endocervical swab for *Chlamydia trachomatis* analysis. During the 5 year period from 1 September 1988 to 1 September 1993, 343 consecutive patients were eligible for

Correspondence: MPM Burger, Department of Obstetrics and Gynaecology, Section Oncological Gynaecology, University Hospital, Hanzeplein 1, NL-9713 GZ Groningen, The Netherlands
Received 23 March 1995; revised 29 September 1995; accepted 19 October 1995

participation in the study. Twenty-three patients were excluded for the following reasons: two patients did not want to be involved in the study, two patients had insufficient command of Dutch or English, four patients were pregnant at the time of colposcopy and 15 patients were not treated in accordance with the study protocol (i.e. no cervical scrape for HPV analysis, or no biopsy, or no treatment when the biopsy disclosed cervical intraepithelial neoplasia). Finally, a benign change or (micro)invasive carcinoma was histologically found in 41 and 14 patients respectively. Therefore, 265 newly diagnosed patients with CIN were included in the study. Some of these patients were also included in previous studies on HPV and CIN (Burger *et al.*, 1993, 1995).

### *Questionnaire*

Using a structured questionnaire we asked the women to state the mean number of cigarettes they were currently smoking per day, their age at first sexual intercourse (sexarche), their lifetime number of sexual partners and whether they had ever had a sexually transmitted disease or genital warts. All the women were told beforehand that the questionnaire comprised some intimate questions and that they were not obliged to answer.

### *Microbiological analyses*

Firstly, an endocervical swab was taken for *Chlamydia trachomatis* analysis. Within 4 h of collection this specimen was inoculated on cycloheximide-treated McCoy cell monolayers on coverslips, as described by Ripa and Mårdh (1977). Detection of *Chlamydia trachomatis* inclusions was performed after 48 h using fluorescein-conjugated monoclonal antibodies (MikroTrak Culture Confirmation, Syva, Palo Alto, CA, USA).

Secondly, after the swab for *Chlamydia trachomatis* culture had been taken the cervix was scraped with the blunt and pointed end of a wooden cervical spatula and with an endocervical brush. The scraped cells were suspended in 5 ml phosphate-buffered saline, pH 7.2, supplemented by merthiolate 1:10 000 v/v. The cell suspension was sent to the laboratory and processed the following morning. The samples were analysed for the presence of HPV with the use of a general primer-mediated polymerase chain reaction (Snijders *et al.*, 1990) and type-specific primer-mediated polymerase chain reactions for the presence of HPV types 6, 11, 16, 18, 31 and 33 separately (Melchers *et al.*, 1989; Claas *et al.*, 1989). If the general primer-mediated reaction was positive but the reactions for types 6, 11, 16, 18, 31 and 33 were negative, the type remained unknown. The laboratory staff were unaware of the histological reports.

### *Morphological examination*

Four weeks after the cervix had been scraped we took representative colposcopically directed biopsy samples of atypical epithelium. If CIN of any grade was diagnosed we subsequently excised the whole transformation zone by loop electrosection or cold knife conisation. Diathermic loop excision was used if the squamocolumnar junction could be visualised entirely and did not extend up into the canal for more than 5 mm from the anatomical os externum. The details of the technique have been described previously (Burger and Hollema, 1993).

Cervical neoplasia was diagnosed and graded according to the criteria of the World Health Organization (Poulsen *et al.*, 1975). The cervical neoplasia was classified according to the most severe lesion found by histological examination.

### *Statistical analysis*

To test for a significant difference between 2 and >2 groups of patients with regard to quantitative variables we used the Mann–Whitney *U*-test and the Kruskal–Wallis test respectively. To test for a significant difference between groups with regard to a qualitative variable, we used the $\chi^2$ test. These non-parametric tests were performed using the SYSTAT software package (Wilkinson, 1990). The $\chi^2$ test for trend and the logistic regression analysis were performed using the EGRET software package (EGRET, 1992). To assess the correlation between two variables with a continuous scale we calculated Spearman's rank correlation coefficient using SYSTAT. The 95% confidence interval of this coefficient was calculated with the use of the CIA software package (Gardner *et al.*, 1991). *P*-values of less than 0.05 were considered to be significant.

### Results

### *The HPV types and the histological diagnoses*

Table I shows the HPV types detected in relation to the different histological diagnoses. HPV was found in 18 (47%) out of the 37 patients with CIN I, in 33 (69%) out of 48 patients with CIN II and in 154 (86%) out of the 180 patients with CIN III. Thus the prevalence of HPV increased with the severity of the neoplastic lesion ($\chi^2$=26.20, d.f.=1, $P$<0.001; test for trend).

The median age of the patients was 35.0 years (interquartile range 29–39 years). No difference was found between the three groups of histological diagnoses with regard to age distribution ($\chi^2$ =1.76, d.f.=2, $P$=0.41; Kruskal–Wallis test).

### *Determinants for the presence of HPV*

Table II shows the results of the comparison between HPV-positive (all types) and HPV-negative patients with regard to the possible determinants. The group of HPV-positive women differed significantly from the group of women without HPV with respect to current age, the lifetime number of sexual partners and the proportion of smokers. The differences with regard to the median value of age and the lifetime number of sexual partners were small: 2 years and one partner respectively. In the smokers the number of cigarettes smoked per day did not differ between the groups ($P$=1.00; Mann–Whitney *U*-test). No relation was found between the presence of HPV and age at first sexual intercourse, a past history of venereal disease, a past history of genital warts and current infection with *Chlamydia trachomatis*.

We used 2×*k*-table analysis and logistic regression to appraise the relations between the presence of HPV and current age, smoking behaviour and the lifetime number of sexual partners. Table III shows the results of the analysis. The proportion of women with HPV decreased significantly

**Table I** The HPV types in relation to the CIN grade

| *HPV type (s)* | *CIN I* | *CIN II* | *CIN III* | *Totals* |
|---|---|---|---|---|
| No HPV | 19 | 15 | 26 | 60 |
| 6/11 | 1 | | | 1 |
| 6/11, 16 | | 1 | 1 | 2 |
| 6/11, 16, 31 | | | 1 | 1 |
| 16 | 4 | 11 | 93 | 108 |
| 16, 18 | 1 | 2 | 3 | 6 |
| 16, 31 | | | 3 | 3 |
| 16, 33 | | | 1 | 1 |
| 18 | 5 | 3 | 11 | 19 |
| 18, 31 | | 1 | | 1 |
| 18, 31, 33 | | | 1 | 1 |
| 18, 33 | 2 | | | 2 |
| 31 | | 5 | 13 | 18 |
| 31, 33 | | 1 | 1 | 2 |
| 33 | | 1 | 8 | 9 |
| Unknown type | 5 | 8 | 18 | 31 |
| Totals | 37 | 48 | 180 | 265 |

as the current age increased ($\chi^2=6.59$, d.f. = 1, $P=0.01$; test for trend). Compared with women under 30 years of age, the odds ratio decreased to 0.35 for women who were 40 years or older. Each year of life conferred a 4% decrease in risk (odds ratio 0.96 with 0.92 and 1.00 as the limits of the 95% confidence interval) when age was analysed as a continuous variable.

The proportion of women with HPV increased significantly as the number of cigarettes smoked a day increased ($\chi^2=10.31$, d.f. = 1, $P=0.001$; test for trend). Compared with women who did not smoke the point estimate of the odds ratio increased to 3.79 in women who smoked more than 20 cigarettes a day. Each cigarette produced a 5% increase in the risk for HPV when the number of cigarettes a day was analysed as a continuous variable.

In the logistic regression analysis age at diagnosis and the number of cigarettes smoked per day could be treated as continuous variables. By contrasting likelihood values it became evident that the descriptive power of the logistic models did not change if these factors were converted to categorical variables. However, treating the lifetime number of sexual partners as a continuous variable obscured the predictive information it contained. The point estimate of the proportion of HPV-positive women among those with one partner and those with two partners in their lifetime was 23/39 (60.0%) and 18/27 (66.7%) respectively. At the point of three partners or more the proportion of HPV-positive women levelled off. The point estimate of the proportion of HPV-positive women who had had ⩾3 partners was 156/189 (82.5%). Therefore, in the logistic models the lifetime number of sexual partners was treated as a categorical variable with three classes (1, 2 and ⩾3). Compared with women with one

**Table II** Distribution of selected determinants in CIN patients according to the presence or absence of HPV (all types)

| Factor | HPV Present (n=205) | HPV Absent (n=60) | P-value |
|---|---|---|---|
| Age (years) | | | |
| Median | 34 | 36 | 0.02[a] |
| Interquartile range | 29–38 | 32–41 | |
| Sexarche (years) | | | |
| Median | 17 | 17.5 | 0.27[a] |
| Interquartile range | 16–19 | 16–19 | |
| Lifetime number of partners | | | |
| Median | 4 | 3 | 0.02[a] |
| Interquartile range | 3–10 | 1–10 | |
| Past hisory of venereal disease | | | |
| Number (%) | 37 (18.0%) | 9 (15.0%) | 0.72[b] |
| Past history of genital warts | | | |
| Number (%) | 23 (11.2%) | 4 (6.7%) | 0.43[b] |
| Current smoking | | | |
| Number (%) | 145 (70.7%) | 29 (48.3%) | 0.002[b] |
| *Chlamydia trachomatis* present | | | |
| Number (%) | 3 (1.5) | 1 (1.7) | – |

[a]Mann–Whitney *U*-test. [b]$\chi^2$ test for two groups with Yates' correction.

**Table III** Odds ratios for HPV infection by current age, number of cigarettes smoked per day and lifetime number of sexual partners in patients with CIN.

| Variable | Proportion with HPV infection (%) | Odds ratio (95% confidence interval) for the HPV infection: Crude | Adjusted[a] |
|---|---|---|---|
| Age (years) | | | |
| ⩽29 | 60/71 (84.5) | 1 (reference) | 1 (reference) |
| 30–34 | 48/59 (81.4) | 0.80 (0.32–2.00) | 0.80 (0.31–2.10) |
| 35–39 | 59/77 (76.6) | 0.60 (0.26–1.38) | 0.71 (0.30–1.68) |
| ⩾40 | 38/58 (65.5) | 0.35 (0.15–0.81) | 0.45 (0.18–1.06) |
| Trend per year | | 0.96 (0.92–1.00) ($P=0.03$) | 0.97 (0.94–1.01) ($P=0.21$) |
| No. of cigarettes smoked per day | | | |
| 0 | 60/91 (65.9) | 1 (reference) | 1 (reference) |
| 1–10 | 40/50 (80.0) | 2.07 (0.91–4.68) | 1.71 (0.73–4.02) |
| 11–20 | 61/74 (82.4) | 2.42 (1.16–5.08) | 1.88 (0.85–4.17) |
| ⩾21 | 44/50 (88.0) | 3.79 (1.45–9.86) | 3.11 (1.16–8.29) |
| Trend per cigarette | | 1.05 (1.01–1.08) ($P=0.003$) | 1.04 (1.01–1.07) ($P=0.02$) |
| Lifetime number of sexual partners[b] | | | |
| 1 | 23/39 (60.0) | 1 (reference) | 1 (reference) |
| 2 | 18/27 (66.7) | 1.39 (0.50–3.87) | 1.23 (0.43–3.50) |
| ⩾3 | 156/189 (82.5) | 3.29 (1.57–6.89) | 2.58 (1.19–5.62) |
| Trend per category | | 1.85 (1.29–2.66) ($P<0.001$) | 1.64 (1.13–2.40) ($P=0.01$) |

[a]The estimates for each of the three factors are adjusted for the two other factors through logistic regression. [b]Data missing for ten cases.

sexual partner in their lifetime the point estimate of the odds ratio increased significantly to 3.29 in women with three or more partners.

We analysed whether smoking behaviour, age at diagnosis and the lifetime number of sexual partners are interrelated. In the group of current smokers the median age was significantly lower than in the group of non-smokers: 32.5 (interquartile range 29–38) years and 35.0 (interquartile range 31.5–41) years respectively ($P=0.001$, Mann–Whitney $U$-test). In the groups of smokers and non-smokers the median lifetime number of sexual partners was four (interquartile range 3–10) and four (interquartile range 2–10) respectively. Although the median values are similar, statistically the smokers have had more partners ($P=0.05$, Mann–Whitney $U$-test). Spearman's rank correlation coefficient between the current age and the lifetime number of sexual partners was −0.21 with a 95% confidence interval from −0.32 to −0.09. Therefore, there was a significantly negative relation between current age and the lifetime number of sexual partners. We conclude that smoking behaviour, current age and the lifetime number of sexual partners are interrelated.

We subsequently performed a multivariate logistic regression analysis. Table III shows that the number of cigarettes smoked per day and the lifetime number of sexual partners are independent significant determinants for the presence of HPV. The inverse association between age and the presence of HPV disappeared after adjustment had been made for smoking and the lifetime number of partners.

Finally, we analysed whether the three variables that we had identified were also associated with HPV in women with CIN II or CIN III. For this purpose we excluded patients with CIN I from the following analysis. The median age (with limits of the interquartile interval) was 35 (29.5–38) years and 36 (32–40) years in the HPV-positive and HPV-negative patients with CIN II–III respectively ($P=0.12$; Mann–Whitney $U$-test). The median lifetime number (with limits of the interquartile range) of sexual partners was 4 (3–10) and 3 (1–8.5) in the HPV-positive and HPV-negative patients with CIN II–III respectively ($P=0.04$; Mann–Whitney $U$-test). The proportion of smokers was 132/187 (70.6%) and 22/41 (53.7%) in the HPV-positive and HPV-negative patients with CIN II–III respectively ($\chi^2=3.66$, d.f. = 1, $P=0.06$; $\chi^2$-test with Yates' correction). We conclude that the results remain largely unaltered when the analysis was restricted to women with CIN II or CIN III.

### Discussion

We found that HPV-positive and HPV-negative CIN patients differed significantly with respect to their smoking behaviour and the lifetime number of sexual partners. We also performed the analysis on patients who were diagnosed with CIN II or CIN III because it has been demonstrated that benign infectious or reactive changes are difficult to discriminate from CIN I (Ismail *et al.*, 1989). Most studies have found that CIN II and CIN III are similar with regard to their associated HPV types and DNA ploidy (Wright and Kurman, 1994). Practically no differences were found compared with the analysis including all women. As women with CIN II and CIN III constituted 228 (86%) of the 265 patients studied, we did no expect that the results would differ substantially. Our findings strongly favour the existence of HPV-negative cervical neoplasia and it is noteworthy that the determinants of the occurrence of HPV in patients with cervical neoplasia show striking similarities to those in other (non-oncological) populations.

Our study group included a comparatively high proportion of women with CIN II or CIN III. We applied stringent cytological ground for entry into the study group. All of the patients had either two mildly or moderately dyskaryotic cervical smears or one severely dyskaryotic smear. In addition, we studied the complete transformation zone because we performed loop electrosection or cold knife conisation. In a previous study we demonstrated that the biopsy diagnosis tends to underestimate the true severity of the disease (Burger and Hollema, 1993).

We found HPV (all types) in 69% and 86% of the patients with CIN II and CIN III, respectively. A limited number of other investigators have reported higher frequencies and some claim that all high-grade CIN lesions harbour HPV (reviewed by Walboomers *et al.*, 1994). Estimates of the prevalence of genital HPV infection depend on the population investigated and the HPV assay used (Guerrero *et al.*, 1992; Schiffman, 1992). Quality assurance programmes have indicated that PCR test interpretation should be done with great care in order to avoid false-positive (and false-negative) assessments (Quint *et al.*, 1995). The probability of detecting HPV is increased if there is ample clinical material available. It has been demonstrated that the proportion of HPV-positive women increases with increasing quantities of cellular DNA in cytological samples (Woodman, 1994). In our study the same set of primers was used throughout and the laboratory staff were unaware of the histological reports.

After adjustment for age and the lifetime number of sexual partners we found a significant independent effect of smoking on the occurrence of HPV. This finding is in accordance with our previous report regarding a significant dose–response relation between the number of cigarettes smoked per day and the occurrence of oncogenic HPV in the cervix of women with cytological abnormalities (Burger *et al.*, 1993). An effect of smoking on the occurrence of HPV, adjusted for the number of sexual partners and age, has also been demonstrated in a case–control study by Kataja *et al.* (1993).

After adjustment for current smoking behaviour and age we found a significant relation between the lifetime number of sexual partners and the presence of HPV. In the linear logistic model the predictive information from the lifetime number of sexual partners was optimised by using three categories: 1, 2 and $\geqslant 3$. If a woman had had three partners, a further increase in the number of partners did not affect the probability that she would be diagnosed with a cervical HPV infection. A relation between the lifetime number of sexual partners and HPV infection, adjusted for smoking behaviour and age, has also been demonstrated by Kataja *et al.* (1993) and Bauer *et al.* (1993). We emphasise that adjustment should be made for smoking if the impact of sexual behaviour on the occurrence of the HPV infection is studied. This follows from the fact that we demonstrated an association between the lifetime number of male sexual partners and smoking behaviour, as has also been done in a study on women attending a university health service (Ley *et al.*, 1991) and on women attending a sexually transmitted diseases clinic (Willmott, 1992).

It is commonly assumed that a comparatively increased number of sexual partners indicates the involvement of a sexually transmittable agent. However, there are also several arguments for non-sexual transmission routes (Woodman, 1994). Our inability to demonstrate a relation between HPV infection and sexually transmitted diseases contributes to the doubts about the importance of sexual transmission. We could not detect an association between the occurrence of HPV and a past history of venereal disease, a past history of genital warts (which are associated with the 'benign' HPV types of 6 and 11) or current cervical *Chlamydia trachomatis* infection. Owing to the fact that several sexually transmitted diseases frequently occur together one would expect to find an association between the presence of HPV and sexually transmittable agents. Other investigators did not find an association between the occurrence of HPV and *Chlamydia trachomatis* in women attending an STD clinic (Claas *et al.*, 1992).

In the univariate analysis we found an inverse relation between the occurrence of HPV and age. This age dependency of the HPV infection has been reported in groups of healthy women (De Villiers *et al.*, 1992; Melkert *et al.*, 1993) and in cervical carcinoma patients (Higgins *et al.*, 1991). All the studies showed that the younger the patient, the higher the chance of detecting HPV. These findings indicate that age should also be considered as a confounder in comparative studies. The prevalence of HPV in healthy young females is high. Dutch investigators, who used a general primer-mediated polymerase chain reaction, detected cervical HPV infection in 25% of the women aged 20–25 years who underwent a routine check-up by their general practitioner because they were using oral contraceptives (Melkert *et al.*, 1993). This figure represents the result of a single determination and repeated determinations at given time intervals are known to result in much higher figures (Schneider and Koutsky, 1992). The combined epidemiological evidence is compatible with the notion that the majority of young people will acquire a genital HPV infection, either by a sexual or non-sexual route. Apparently, most HPV infections resolve spontaneously with increasing age. A small percentage of women will suffer from persistent infections with neoplasia as a possible consequence if the HPV type concerned is oncogenic. Nevertheless, our study provides strong arguments in favour of a non-HPV-related aetiological route for cervical neoplasia.

### References

BAUER HM, HILDESHEIM A, SCHIFFMAN MH, GLASS AG, RUSH BB, SCOTT DR, CADELL DM, KURMAN RJ AND MANOS MM. (1993). Determinants of genital human papillomavirus infection in low-risk women in Portland, Oregon. *Sex. Transm. Dis.*, **20**, 274–278.

BERGERON C, BARRASSO R, BEAUDENON S, FLAMANT P, CROISSANT O AND ORTH G. (1992). Human papillomaviruses associated with cervical intraepithelial neoplasia. Great diversity and distinct distribution in low- and high-grade lesions. *Am. J. Surg. Pathol.*, **16**, 641–649.

BURGER MPM AND HOLLEMA H. (1993). The reliability of the histologic diagnosis in colposcopically directed biopsies. A plea for LETZ. *Int. J. Gynecol. Cancer*, **3**, 385–390.

BURGER MPM, HOLLEMA H, GOUW ASH, PIETERS WJLM AND QUINT WGV. (1993). Cigarette smoking and human papillomavirus in patients with reported cervical cytological abnormality. *Br. Med. J.*, **306**, 749–752.

BURGER MPM, HOLLEMA H, PIETERS WJLM AND QUINT WGV. (1995). Predictive value of human papillomavirus type for histological diagnosis of women with cervical cytological abnormalities. *Br. Med. J.*, **310**, 94–95.

CLAAS E, MELCHERS W, VAN DEN LINDEN J, LINDEMAN J AND QUINT W. (1989). Human papillomavirus detection in paraffin embedded cervical carcinomas and their metastases by the polymerase chain reaction. *Am. J. Pathol.*, **135**, 703–709.

CLAAS ECJ, MELCHERS WJG, NIESTERS HGM, VAN MUYDEN R, STOLZ E AND QUINT WGV. (1992). Infections of the cervix uteri with human papillomavirus and *Chlamydia trachomatis*. *J. Med. Virol.*, **37**, 54–57.

DE VILLIERS EM. (1989). Minireview. Heterogeneity of the human papillomavirus group. *J. Virol.*, **63**, 4898–4903.

DE VILLIERS EM, WAGNER D, SCHNEIDER A, WESCH H, MUNZ F, MIKLAW H AND ZUR HAUSEN H. (1992). Human Papillomavirus DNA in women without and with cytological abnormalities: results of a five year follow-up study. *Gynecol. Oncol.*, **44**, 33–39.

EGRET. (1992). *Epidemiological Graphics, Estimation and Testing Package*, version 0.26.6. Statistics and Epidemiology Research Corporation: Seattle.

FRANCO EL. (1994). Meeting report 13th international papillomavirus conference. *Papillomavirus Rep.*, **5**, 183–187.

GARDNER MJ, GARDNER SB AND WINTER PD. (1991). *Confidence Interval Analysis (CIA) Microcomputer Program*. British Medical Journal: London.

GUERRERO E, DANIEL RW, BOSCH FX, CASTELLSAGUE X, MUÑOZ N, GILI M, VILADIU P, NAVARRO C, MARTOS C, ASCUNCE N, GONZALEZ LC, TAFUR L, IZARZUGAZA I AND SHAH KV. (1992). Comparison of Virapap, Southern hybridization, and polymerase chain reaction methods for human papillomavirus (HPV) identification in an epidemiological investigation of cervical cancer. *J. Clin. Microbiol.*, **30**, 2951–2959.

HIGGINS GD, DAVY M, RODER D, UZELIN DM, PHILLIPS GE AND BURRELL CJ. (1991). Increased age and mortality associated with cervical carcinomas negative for human papillomavirus RNA. *Lancet*, **338**, 910–913.

ISMAIL SM, COLCLOUGH AB, DINNEN JS, EAKINS D, EVANS DMD, GRADWELL E, O'SULLIVAN JP, SUMMERELL JM AND NEWCOMBE RG. (1989). Observer variation in histopathological diagnosis and grading of cervical intraepithelial neoplasia. *Br. Med. J.*, **298**, 707–710.

KATAJA V, SYRJÄNEN S, YLISKOSKI M, HIPPELÄNEN M, VÄYRYNEN M, SAARIKOSKI S, MÄNTYJÄRVI R, JOKELA V, SALONEN JT AND SYRJÄNEN K. (1993). Risk factors associated with cervical human papillomavirus infections: a case control study. *Am. J. Epidemiol.*, **138**, 735–745.

LEY C, BAUER HM, REINGOLD A, SCHIFFMAN MH, CHAMBERS JC, TASHIRO CJ AND MANOS MM. (1991). Determinants of genital human papillomavirus infection in young women. *J. Natl Cancer Inst.*, **83**, 997–1003.

LUNGU O, SUN XW, FELIX J, RICHART RM, SILVERSTEIN S AND WRIGHT TC. (1992). Relationship of human papillomavirus type to grade of cervical intraepithelial neoplasia. *JAMA*, **267**, 2493–2496.

MELCHERS W, VAN DEN BRULE A, WALBOOMERS J, DE BRUIN M, BURGER M, HERBRINK P, MEIJER C, LINDEMAN J AND QUINT W. (1989). Increased detection rate of human papillomavirus in cervical scrapes by the polymerase chain reaction as compared to modified FISH and Southern-blot analysis. *J. Med. Virol.*, **27**, 329–335.

MELKERT PWJ, HOPMAN E, VAN DEN BRULE AJC, RISSE EKJ, VAN DIEST PJ, BLEKER OP, HELMERHORST T, SCHIPPER MEI, MEIJER CJLM AND WALBOOMERS JMM. (1993). Prevalence of HPV in cytomorphologically normal cervical smears, as determined by the polymerase chain reaction, is age-dependent. *Int. J. Cancer*, **53**, 1–5.

POULSEN HE, TAYLOR CW AND SOBIN LH. (1975). *Histological Typing of Female Genital Tract Tumours*. World Health Organization: Geneva.

QUINT WGV, HEIJTINK RA, SCHIRM J, GERLICH WH AND NIESTERS HGM. (1995). Reliability of methods for hepatitis B virus DNA detection. *J. Clin. Microbiol.*, **33**, 225–228.

RICHART RM. (1973). Cervical intraepithelial neoplasia: a review. In *Pathology Annual*, Sommers SC (ed.) pp. 301–328. Appleton-Century-Crofts: New York.

RIOU G, FAVRE M, JEANNEL D, BOURHIS J, DE DOUSSAL V AND ORTH G. (1990). Association between poor prognosis in early-stage invasive cervical carcinomas and non-detection of HPV DNA. *Lancet*, **335**, 1171–1174.

RIPA KT AND MÅRDH P-A. (1977). Cultivation of *Chlamydia trachomatis* in cycloheximide-treated McCoy cells. *J. Clin. Microbiol.*, **6**, 328–331.

SCHIFFMAN MH. (1992). Validation of HPV hybridization assays: correlation of filter *in situ*, dot blot and PCR with Southern blot. In *The Epidemiology of Human Papillomavirus and Cervical Cancer*, IARC Scientific Publications No. **119**, Muñoz N, Bosch FX, Shah KV, Meheus A. (eds.) pp. 169–179. IARC: Lyon.

SCHIFFMAN MH. (1994). Epidemiology of cervical human papillomavirus infections. In *Human Pathogenic Papillomaviruses*. pp. 55–81. Zur Hausen H, (ed.) Springer: Berlin.

SCHNEIDER A AND KOUTSKY LA. (1992). Natural history and epidemiological features of genital HPV infection. In *The Epidemiology of Human Papillomavirus and Cervical Cancer*, IARC scientific publications No. **119** Muñoz N, Bosch FX, Shah KV, Meheus A. (eds.) pp. 25–52. IARC: Lyon.

SNIJDERS PJF, VAN DEN BRULE A, SCHRIJNEMAKERS H, SNOW G, MEIJER CJLM AND WALBOOMERS JMM. (1990). The use of general primers in the PCR permits the detection of a broad spectrum of human papillomavirus infections. *J. Gen. Virol.*, **72**, 2781–2786.

VAN DEN BRULE AJC, SNIJDERS PJF, GORDIJN RLJ, BLEKER OP, MEIJER CJLM AND WALBOOMERS JMM. (1990). General primer-mediated polymerase chain reaction permits the detection of sequenced and still unsequenced human papillomavirus genotypes in cervical scrapes and carcinomas. *Int. J. Cancer*, **45**, 644–649.

WALBOOMERS JMM, DE RODA HUSMAN A-M, VAN DEN BRULE AJC, SNIJDERS PJF AND MEIJER CJLM. (1994). Detection of genital human papillomavirus infections: critical review of methods and prevalence studies in relation to cervical cancer. In *Human Papillomaviruses and Cervical Cancer. Biology and Immunology*. Stern PL and Stanley MA. (eds.) pp. 41–71. Oxford University Press: Oxford.

WILKINSON L. (1990). *SYSTAT: the System for Statistics*. SYSTAT: Evanston, IL.

WILLMOTT FE. (1992). Current smoking habits and genital infections in women. *Int. J. STD AIDS*, **3**, 329–331.

WOODMAN C. (1994). Epidemiology of HPV and cervical cancer. In *Human Papillomaviruses and Cervical Cancer. Biology and Immunology, Stern PL and Stanley MA*. (eds.) pp. 72–91. Oxford University Press: Oxford.

WRIGHT Jr TC AND KURMAN RJ. (1994). A critical review of the morphologic classification systems of preinvasive lesions of the cervix: the scientific basis for shifting the paradigm. *Papillomavirus Rep.*, **5**, 175–182.